# A Real-Life Reproducibility Assessment for NMR Metabolomics †

**DOI:** 10.3390/diagnostics12030559

**Published:** 2022-02-22

**Authors:** Cristina Stavarache, Alina Nicolescu, Cătălin Duduianu, Gabriela Liliana Ailiesei, Mihaela Balan-Porcăraşu, Mihaela Cristea, Ana-Maria Macsim, Oana Popa, Carmen Stavarache, Anca Hîrtopeanu, Lucica Barbeş, Raluca Stan, Horia Iovu, Calin Deleanu

**Affiliations:** 1 “C.D. Nenitescu” Centre of Organic Chemistry, Romanian Academy, 060023 Bucharest, Romania; crisstavarache@gmail.com (C.S.); duduxian94@gmail.com (C.D.); oana.popa926@gmail.com (O.P.); stavarachec@yahoo.com (C.S.); ahirtopeanu@yahoo.com (A.H.); 2 Advanced Polymer Materials Group, University Politehnica of Bucharest, 011061 Bucharest, Romania; horia.iovu@upb.ro; 3 “Petru Poni” Institute of Macromolecular Chemistry, Romanian Academy, 700487 Iasi, Romania; gdarvaru@icmpp.ro (G.L.A.); mihaela.balan@icmpp.ro (M.B.-P.); cristea.mihaela@icmpp.ro (M.C.); macsim.ana@icmpp.ro (A.-M.M.); 4 Faculty of Applied Chemistry and Material Science, University Politehnica of Bucharest, 011061 Bucharest, Romania; raluca.stan@upb.ro; 5 Department of Chemistry and Chemical Engineering, “Ovidius” University of Constanta, 900527 Constanta, Romania

**Keywords:** metabolomics, NMR, reproducibility, interlaboratory trial, quality control

## Abstract

Nuclear magnetic resonance (NMR) metabolomics is currently popular enough to attract both specialized and non-specialized NMR groups involving both analytical trained personnel and newcomers, including undergraduate students. Recent interlaboratory studies performed by established NMR metabolomics groups demonstrated high reproducibility of the state-of-the-art NMR equipment and SOPs. There is, however, no assessment of NMR reproducibility when mixing both analytical experts and newcomers. An interlaboratory assessment of NMR quantitation reproducibility was performed using two NMR instruments belonging to different laboratories and involving several operators with different backgrounds and metabolomics expertise for the purpose of assessing the limiting factors for data reproducibility in a multipurpose NMR environment. The variability induced by the operator, automatic pipettes, NMR tubes and NMR instruments was evaluated in order to assess the limiting factors for quantitation reproducibility. The results estimated the expected reproducibility data in a real-life multipurpose NMR laboratory to a maximum 4% variability, demonstrating that the current NMR equipment and SOPs may compensate some of the operator-induced variability.

## 1. Introduction

Metabolomics has become an important topic in a wide range of scientific areas, including medicine, pharmacology, nutrition and metabolism, food sciences and environmental research.

To date, there are only two analytical techniques, namely MS and NMR spectroscopy, suitable for large metabolomic screenings and for epidemiological studies. The main advantages of the NMR method are that it provides direct quantitative information on selected markers (even in the absence of a standard) and, also, it provides an untargeted global biochemical profile with minimum sample preparation.

Metabolomics involves the simultaneous detection of a large number of compounds from a living system or a naturally occurring ecosystem. As metabolomics requires the processing of large numbers of multiple parameters, the reproducibility of data for statistical purposes is a key issue.

Quantitation accuracy [1,2] and reproducibility [3,4] in NMR studies are important issues and have been subjected to several interlaboratory quality control (QC) tests long before the NMR metabolomics emerged. These issues continue to be highly important for the chemical and pharma industry [5,6,7], factors affecting the accuracy of quantitation techniques being extensively explored [7,8,9,10,11,12,13,14]. Currently, reproducibility is a hot topic driven by various biomedical [15,16,17,18,19,20] and food applications [21,22,23,24,25,26,27,28,29]. With the advent in NMR-based human phenotyping, as well as urine and plasma-based medical diagnosis and health status monitoring, several large groups have become well-established in NMR metabolomics, and recent studies have proven the impressive interlaboratory reproducibility of NMR spectra when employing trained personnel and the latest industry standard solutions [16,17,30].

Metabolomics is currently a well-established topic both in education and in a wide range of research areas. Thus, in addition to dedicated metabolomics groups, NMR metabolomics has penetrated many groups with nonexclusive or even marginal metabolomics interests. Our group is a good example where interests in various NMR topics coexist. Thus, we are involved in advanced structure elucidation studies using multinuclear NMR [31] and materials characterization [32], as well as human [33,34,35,36,37,38], animal [39] and vegetal metabolomics [40,41]. We currently use four what we call “multipurpose” NMR instruments both for our own research interests and as an NMR service for many groups around the country. In terms of NMR metabolomics, we have long-term expertise [33,34,35,36,37,38,39,40,41], also being involved in one of the first multicenter trials for plasma quantitation by NMR [42]. Over the years, we have been using and exchanging five generations of NMR instruments, currently with the latest generation in use. For human and animal metabolomics, we have only four operators preparing the samples, while 10 operators in the group are preparing samples for all the other topics, and there are also occasional students and open access researchers preparing samples and running NMR experiments. We assume that this type of multipurpose NMR laboratory is typical for many other research groups. For the present study, we used two NMR instruments located in two different towns, and we also involved operators from outside the NMR group.

The purpose of the present study was to evaluate the NMR reproducibility for metabolomics in a “real-life” situation when combining both industry standard NMR solutions and multipurpose NMR equipment and to compare the reliability of NMR metabolomics data when involving both dedicated NMR operators (including researchers and technicians) and chemistry users from outside the NMR group (including researchers and students).

## 2. Materials and Methods

Bruker Avance Neo and Bruker Avance III HD 600 MHz NMR instruments (Bruker Biospin, Ettlingen, Germany) were used. Both instruments were equipped with 5-mm z-gradient inverse detection (BBI) probes. TopSpin v.4.0.8 and 3.5PL6 software were used for controlling the spectrometers and data processing. Spectra were recorded with pulse sequences and parameters as delivered with the Bruker Biospin IVDr methods V.1.0 and V.2.0, Bruker Biospin, Ettlingen, Germany), which allowed the fast acquisition of ^1^H NMR spectra using 32 scans, a 90° pulse, 4-s relaxation delay with simultaneous CW irradiation and 2.7-s acquisition time with an ERETIC type of signal as the quantitation reference and J-Resolved NMR spectra for signal assignment support [30]. Spectra were recorded at 300 K. Before each set of samples, a QC check was performed with three standard samples in order to check and correct, when necessary, the temperature regulation, the ERETIC quantification and the shim for magnetic field homogeneity. 

Eight adjustable 0.1–1.0-mL and 0.01–0.1-mL automatic pipettes Gilson Pipetman (Gilson Inc., Middletown, WI, USA), Thermo Fisher Finnpipette (Thermo Fisher Scientific, Vantaa, Finland) and Eppendorf Research Plus (Eppendorf SE, Hamburg, Germany) were used as they are, without further calibration checks.

Three balances with 0.1-mg readout and 0.2-mg reproducibility Kern ALJ 120-4 (Kern & Sohn GmbH, Balingen-Frommern, Germany), Partner WAS220 and Precisa LS120A (Precisa GRavimetrics AG, Dietikon, Switzerland) were used as they are, without further calibration checks.

Five-millimeter NMR tubes from four different lots, i.e., two different lots of type 7” Wilmad 507, one lot of type 7” Bruker 500 (Boro500) and one lot of type 4” Bruker SampleJet were used.

D_2_O (99.9% D, EurIsotop, Saint-Aubin, France), absolute ethanol (Chemical Company, Iasi, Romania) and buffer solution consisting of 6-mM sodium 3-(trimethylsilyl)-[2,2,3,3-d4]-1-propionate (TSP)/KH_2_PO_4_/KOH/D_2_O (Bruker Biospin, Ettlingen, Germany) were used as they are.

## 3. Results and Discussion

### 3.1. Estimation of the Operator Induced Variability

In order to estimate the effect of human error on the reproducibility of data, a large number of pipettings and weighings were performed by several operators with different research skills and backgrounds.

Nineteen operators were involved in these experiments, with the following backgrounds: four researchers were involved mainly in NMR structure elucidations, five were undergraduate chemistry students, one was a MS student involved in NMR metabolomics, three were researchers frequently involved in NMR metabolomics, one was a technician involved in chemistry activities, two were researchers involved in organic synthesis, one was a technician occasionally involved in metabolomics and two were entry-level assistant researchers.

**Figure 1:**
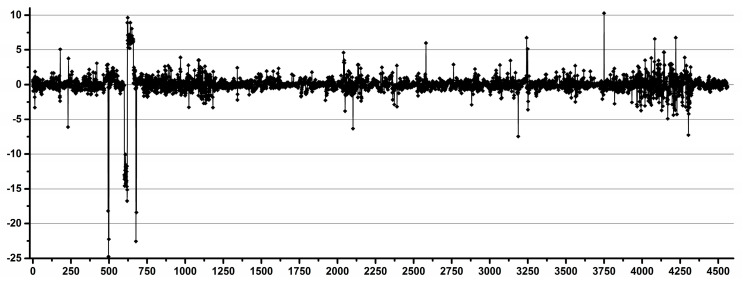
Overview of the individual deviations as a percentage from the averaged values for all pipetting-weighing experiments emphasizing the operator-induced variability.

The experimental design was the following. Each operator performed a total number of 240 pipettings and weighings of distilled water over several days as follows: 60 pipettings and weighings of 0.1 mL by exchanging the pipette tip after each pipetting, 60 pipettings and weighings of 0.1 mL using the same tip for 10 consecutive pipettings and the same experiments repeated for 0.9 mL. A total number of three analytical balances and eight automatic pipettes were used in two laboratories, but each operator used only one balance and one pair of pipettes. In order to minimize the instrumental artefacts and to emphasize the operator’s variability, for each set of the 60 experiments, the average weight was calculated, and for each individual experiment, the deviation was calculated as a percentage, considering the average as the 100% value. Thus, the percentage variations for all 240 experiments for each operator, as well as the total of 4560 experiments performed by all operators, could be combined in the same statistical model. The result is presented as a Shewhart chart in Figure 1, with the number of experiments ordered by operator.

We interpret the results presented in Figure 1 as the operator inducing experimental errors in metabolomics studies involving manual pipetting. There are two important observations: (i) that there are a few outliers (less than 1.7% of all data, i.e., 76 out of 4560 experiments) with over 10% deviations, which may be considered accidents (e.g., occasional distraction of the operator or experimental errors like opening a door or vibrations), and (ii) differences in the distribution of the main core of data for various sets of experiments, which may be attributed to differences in the operator’s performance. The experiments show that 98.3% of the data had a maximum variability of 10% (±5%), regardless of the operator’s qualifications. When looking at individual operators, excluding occasional accidents, the situation is very different: 16 out of 19 operators had less than 4% variability, 15 out of 19 operators had less than 3% variability and 8 out of 19, including all operators involved in the occasional metabolomics sample preparation had less than 2% variability.

In conclusion, with a reasonable selection of operators, one can easily achieve less than 4% human-induced variability.

Thus, all researchers and technicians, regardless of their field of activity, and, also, some of the students performed with less than 4% variability.

**Figure 2:**
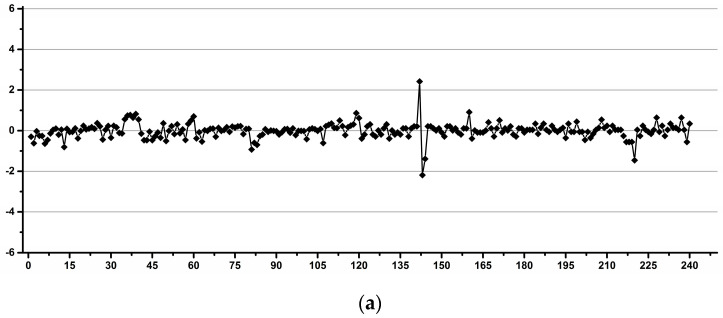

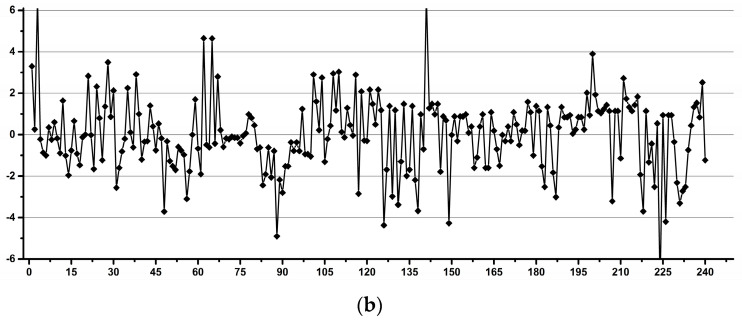
Examples of good (**a**) and low (**b**) reproducibility operators for pipetting.

Figure 2 shows examples of operators with good (a) and low (b) reproducibility during pipetting.

### 3.2. Estimation of the Experimental Induced Variability

In order to estimate the effects of the overall experimental factors, i.e., the electronics stability, the NMR tube variability and the ERETIC signal used for quantification, a large number of NMR tubes was used for recording the spectra of the same solution of ethanol in 10% D_2_O + 90% H_2_O. As we reused most of the high-quality tubes, and, also, as we noticed that even a few new tubes had factory defects, we recorded for all tubes the ^1^H gradient profile in addition to the ^1^H NMR experiment used for quantification. 

The gradient profile [43] indicates if there are scratches or other tube imperfections (or sample inhomogeneities). Such tube imperfections lead to a low water suppression quality with the residual water signal extending over a larger spectral region.

**Figure 3:**
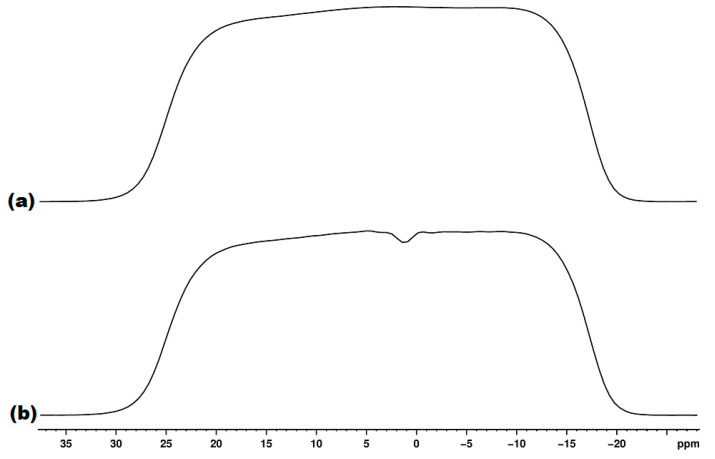
Good gradient profile (**a**) and bad gradient profile (**b**).

**Figure 4:**
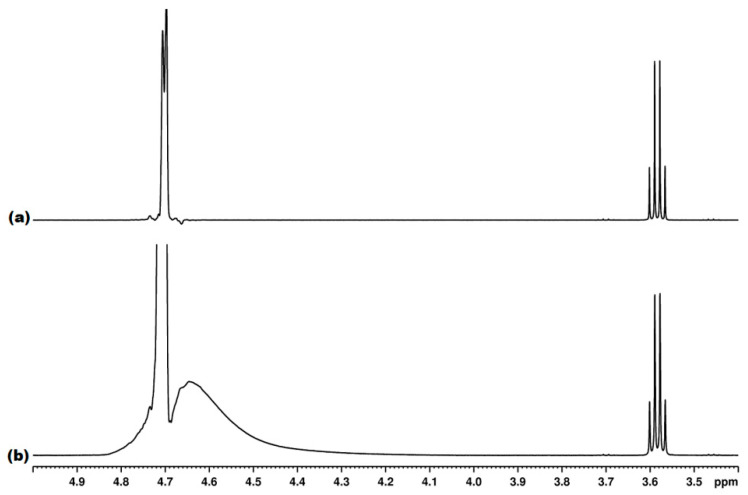
^1^H NMR spectrum with water suppression recorded with good quality tube exhibiting a good gradient profile (**a**) and a low-quality tube with a bad gradient profile (**b**).

Figure 3 exemplifies a high-quality tube with a good gradient profile (a) in comparison with a low-quality tube with a bad gradient profile (b). Figure 4 exemplifies the water suppression result with a good gradient (a) and a bad gradient (b). In the former case, a narrow spectral region of about 0.1 ppm (4.65–4.75 ppm) is affected by the residual water signal, whereas, in the latter case, an almost 10× wider region of about 0.8 ppm (4.05–4.85) is affected by the residual water signal (Figure 4).

All tubes with a bad gradient profile were excluded from the quantification statistics discussed below, and they were also excluded from the laboratory inventory.

The NMR spectra were run in two different laboratories from two different towns over several days. The number of days and runs depended on the capacity of the sample changer. In order to maximize the influence of the tube quality and to minimize the influence of the other instrumental artifacts, for each daily run, the averaged methyl integral was set to 100% and individual variations calculated as the percentage variations.

**Figure 5:**
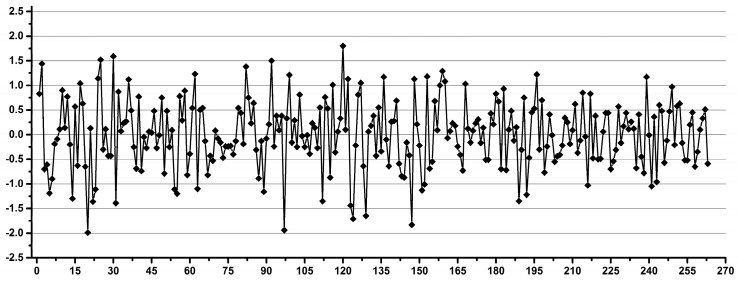
Tube variability of the methyl integral on instrument I1 (600 MHz equipped with 24 positions sample changer) recorded on 16 different days for a total of 263 tubes from the same manufacturer and quality type.

**Figure 6:**
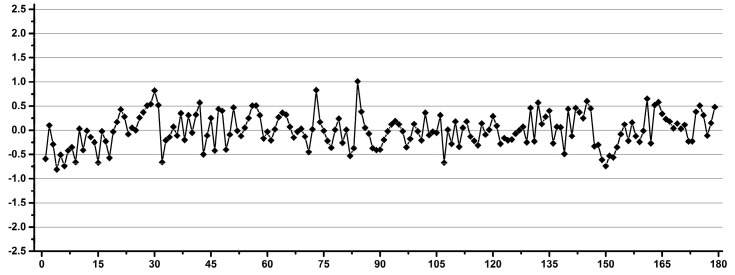
Tube variability of the methyl integral on instrument I2 (600 MHz equipped with 96 positions sample changer) recorded on 3 different days for a total of 179 tubes from the same manufacturer and quality type.

The results for two sets of the same brand and quality of NMR tubes recorded on two NMR instruments at the same magnetic field are presented in Figure 5 and Figure 6.

The results presented in Figure 5 and Figure 6 show an overall variability for all the samples well within 4%, with 97% of all the data below 3% variability (i.e., only 8 out of 263 experiments were higher than 3%). For one instrument, all data were below 2%. As the two instruments were comparable in terms of hardware and software performances (I1 previous to the latest and I2 the latest generation from the same manufacturer), we assumed that the larger variation for one instrument was due to lower room stability conditions (the instrument was not separated from the operator’s area). Additionally, for the same instrument (I1), due to smaller sampler changer capacity, the same number of samples was run over 16 different days, whereas, for the other instrument, the samples were run over 3 different days, which might also induce higher variations in the former case. Of course, the slight difference in electronics performance (although both instruments are IVDr compatible) may play a role in the induced variability.

**Figure 7:**
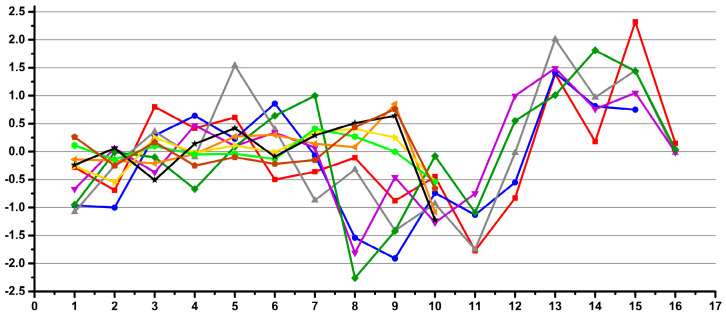
The methyl integral variation for the spectra recorded with the same 10 tubes of the same type repeated several times.

In order to minimize the tube-induced variability and to maximize the influence of the ERETIC and instrument-related factors, 10 tubes (five for each instrument) were run between 10 and 16 times. After each run, the tube was ejected from the magnet in order to be subjected at each rerun to the same temperature equilibration and shim adjustment cycles. The methyl integral was averaged for each tube and the individual variations calculated as a percentage from the averaged integral for each instrument. As it can be seen in Figure 7, the maximum overall variability was 4.5%.

When considering the variability separated by instruments, for instrument I1, the maximum overall variability was 4.5%, with the individual tube variability for five tubes run 16 times each between 2.5 and 4.1%. For instrument I2, the maximum overall variability was 2.1%, with the individual tube variability for five tubes run 10 times each between 1.0 and 1.9%. 

These findings show that the tube-induced variability was the same or below the overall instrument-induced variability, as no improvement was obtained by repeating the experiments with a small number of tubes in comparison with a much larger set of tubes.

**Figure 8:**
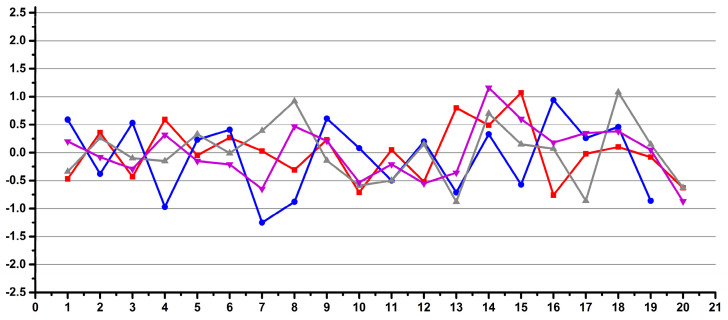
The methyl integral variation for 80 tubes belonging to 4 types when the individual deviations were considered in comparison with the averaged value for the corresponding NMR run. The ERETIC calibration was different for spectra 1–5 and 6–20.

**Figure 9:**
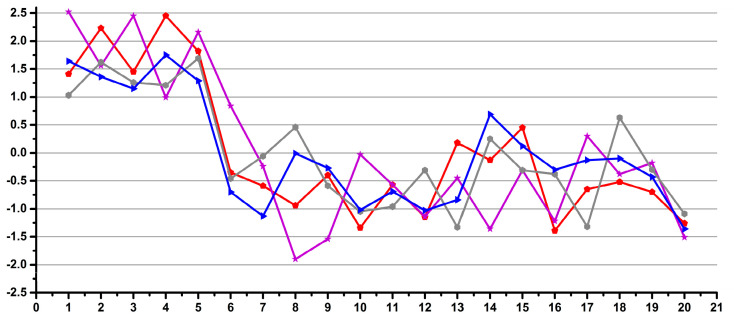
The methyl integral variation for 80 tubes belonging to 4 types when the individual deviations were considered in comparison with the total averaged value for both NMR runs. The ERETIC calibration was different for samples 1–5 and 6–20.

In terms of brand quality, we compared four tube types from two manufacturing sources, three of the same theoretical quality and one of a lower quality. The methyl integral reproducibility as a percentage deviation relative to the average for 20 tubes of each type was within the maximum 2.7% (with over 90% of the data within a 2% variation). The samples were recorded in two different runs over two consecutive days, and the methyl integrals were averaged for each day. The results are presented as a Shewhart chart in Figure 8. Although the spectra were run over two consecutive days with identical laboratory conditions, the ERETIC reference signal (although within QC specifications) was recalibrated before the second run (with a difference of 6 months between the two calibrations). If the methyl integrals were averaged as a total for the two runs, then the individual variations were within 4.4% (with 90% of all the data within a 3% variation) which was consistent with the extended studies for over 442 tubes described above. Thus, in Figure 9, the first five tubes from each type show a small shift in the integral deviation as the integral is measured based on an ERETIC signal calibrated 6 months before recording the spectra, whereas the following 15 integrals for each tube are measured relative to on an ERETIC signal calibrated in the same day with the NMR run. It is obvious that, for each calibration, the integral variation for all the types of tubes is within 3%, and if one considers the variations with ERETIC recalibration, this is within 4.4%. All four studied types of tubes behaved similarly in terms of reproducibility of data. The result confirmed that the tube-induced variability was below the overall instrument-induced variability.

Although the main purpose of the study was to evaluate the reproducibility of the data, at this point, it was also relevant to compare the absolute integral values of the methyl group for each type of tube. Thus, the averaged integral values for the combined runs for the first day ERETIC and for the second day ERETIC calibrations, respectively, were as follows: 65.8/64.6/66.2 for Tubes Type 1, 69.8/68.5/70.3 for Tubes Type 2, 69.4/68.5/69.7 for Tubes Type 3 and 69.5/68.5/69.8 for Tubes Type 4.

If one considers the average of the averages 68.6, then the four types of tubes provided quantitative results of 95.9% (Tubes Type 1), 101.7% (Tubes Type 2), 101.2% (Tubes Type 3) and 101.3% (Tubes Type 4) relative to the overall average.

Thus, depending on tube type, ERETIC-based quantification may lead to absolute differences of up to 5.8%. Thus, as it is expected, one can easily gain about 2% accuracy by using only one type of NMR tube from the same manufacturer in the same metabolomics dataset.

**Figure 10:**
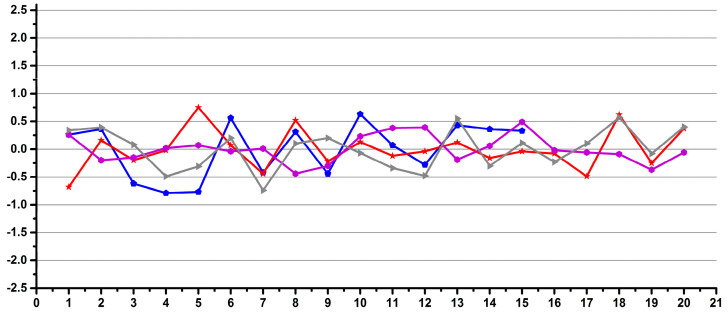
The variation of methyl/methylene integral ratios for 80 tubes belonging to four types of tubes.

In conclusion, regardless of the type of instrument and local stability conditions, and considering that the same types of tubes are used, the overall integral variations for all the tubes were less than 4% for inter-sample comparisons. The intrasample reproducibility (as deviations for methyl/methylene integral ratios) was less than 2% (1.54%), which was, as expected, 50% better than the inter-sample variability, as can be seen in Figure 10.

Thus, the maximum variation for the individual tubes was 1.42, 1.43, 0.93 and 1.06%, respectively, for the series of Tube Types 1–4, and this was, as expected, independent of the external calibration of the ERETIC signal.

### 3.3. Estimation of the Overall Induced Variability for NMR Metabolomics

For the overall induced variability, several operators prepared several samples of the same pool of urine by pipetting the urine and the standard, and the samples were run on two instruments operating at the same magnetic field in two different laboratories from two different towns. 

**Figure 11:**
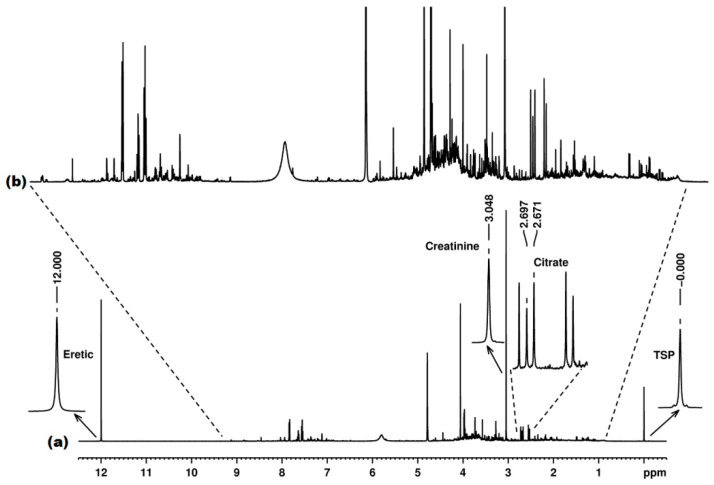
The ^1^H NMR spectrum with water pre-saturation for the urine pool sample recorded at 600 MHz, with details of the signals used in the reproducibility studies (**a**) and the amplified spectrum showing the complexity of signals generated by various metabolites (**b**).

The signals for ERETIC, citrate, creatinine and TSP were processed as absolute and relative integral values. Figure 11 shows the whole ^1^H NMR spectrum of the urine pool used in this study, with details (Figure 11a) of the signals used for reproducibility studies (ERETIC at 12.00 ppm, creatinine at 3.05 ppm, citrate as a doublet centered at 2.68 ppm and TSP at 0.00 ppm).

The same parameters for phase correction and baseline correction were applied to each set of samples (each NMR instrument). For integration, the slope parameters and the same starting/ending frequency values were used for all samples in both sets (both NMR instruments).

In order to evaluate the expected “real-life” reproducibility for metabolomics, we processed the NMR dataset in several ways. Thus, for integral referencing, we used, in separate statistics models, the ERETIC signal (as the external electronic reference), the TSP signal (as the internal operator-introduced reference) and the creatinine signal (as the internal endogenous reference).

In practical metabolomics, each referencing approach has both advantages and drawbacks. Thus, the ERETIC signal has the convenience of not altering/contaminating the sample with external chemicals, and it may also allow faster manual sample preparation by less experienced operators. However, in addition to the instrumental-induced variability, this approach would also be affected by the NMR tubes quality-induced variability.

The TSP signal is the “classical” approach used for both frequency referencing and quantitation referencing in NMR spectroscopy. This approach has the advantage that it reduces the instrumental-induced variability and eliminates the NMR tube-induced variability. However, this approach is the most prone to operator-induced variability, and it relies on well-trained operators for sample preparation.

The internally referenced concentrations to creatinine is a well-established approach for clinical analyses, and it is the least affected by externally induced variability. Thus, referencing all the metabolites to creatinine, for medical samples, or to another naturally occurring endogenous metabolite for other types of samples, it generates models correlated to the natural variability of the reference metabolite, which is either diurnal or has a trend given by factors like age, sex or state of health, and usually, it is the combination of multiple factors affecting both the periodical variability and the trend of evolution of the endogenous reference metabolite.

As mentioned above, we explored the variability induced by all these three referencing approaches using the same NMR spectral dataset.

In order to evaluate the influence of the other experimental factors, we also processed the NMR dataset by normalizing the data according to the operator, the instrument on which they were recorded, the day when they have been recorded and with combinations of such normalizations.

Examples of the relevant influences of all these factors are presented below.

**Figure 12:**
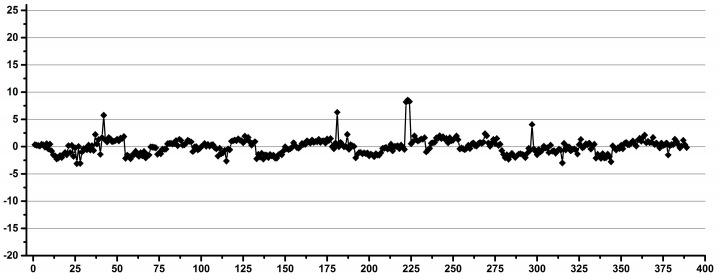
Crn/ERETIC integral variability relative to the averaged integrals for all the samples. The samples are ordered by operator. Only 2.8% of all the data (i.e., 11 out of the total of 389 spectra) has integral variability higher than 5% (±2.5% variability).

**Figure 13:**
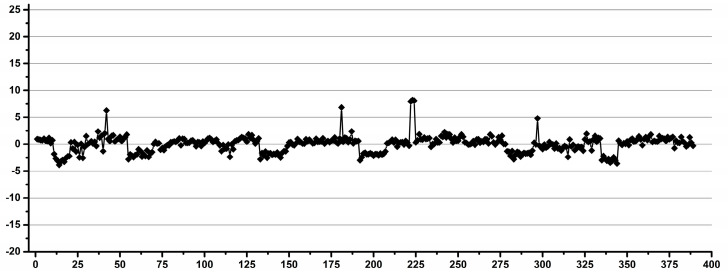
Cit/ERETIC integral variability relative to the averaged integrals for all the samples. Only 3.1% of all the data (i.e., 11 out of the total of 389 spectra) has integral variability higher than 6% (±3% variability). Only 6.4% of all the data, i.e., 25 spectra out of the total of 389 spectra, has integral variability higher than 5% (±2.5% variability).

**Figure 14:**
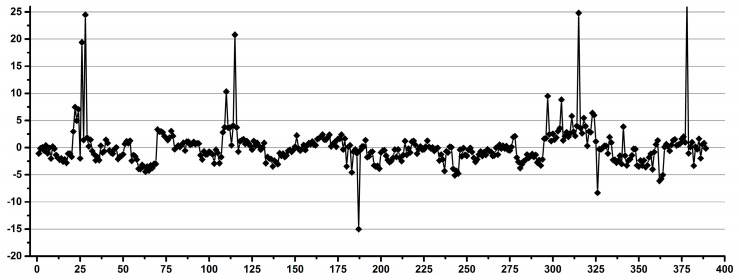
TSP/ERETIC integral variability relative to the averaged integrals for all the samples. Almost 5% (4.9%) of all the data (i.e., 19 out of the total of 389 spectra) has an integral variability higher than 10% (±5% variability).

Figure 12, Figure 13 and Figure 14 present the variability of the integrals for the signals belonging to creatinine (Crn), citrate (Cit) and TSP relative to the integral of the ERETIC signal for the whole NMR dataset (all operators and both NMR instruments).

The Shewhart charts display, on the y-axis, the errors of each individual spectrum relative to the averaged integral of all the samples. On the x-axis of the graphs, each point represents the integral in one individual spectrum ordered by operators and NMR instruments.

**Figure 15:**
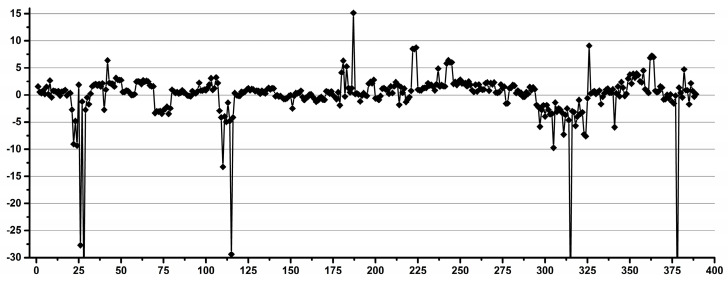
Crn/TSP integral variability relative to the averaged integrals for all the samples. Almost 8% (7.7%) of all the data (i.e., 30 out of the total of 389 spectra) has an integral variability higher than 10% (±5% variability).

**Figure 16:**
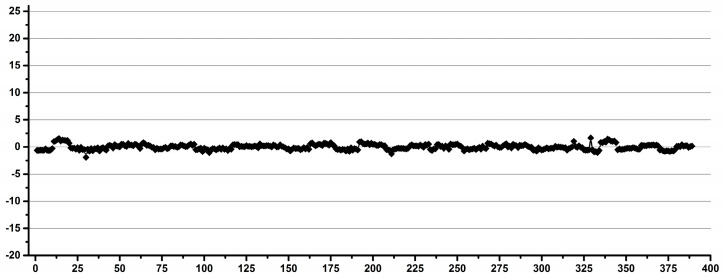
Cit/Crn integral variability relative to the averaged integrals for all samples. All the data have an integral variability below 4% (±2% variability). Only 0.8% of all the data (i.e., 3 out of the total of 389 spectra) has an integral variability higher than 3% (±1.5% variability), and only 4.9% of all the data (i.e., 19 out of the total of 389 spectra) has an integral variability higher than 2% (±1% variability).

Similarly, Figure 15 and Figure 16 present the same trends for integrals of Crn relative to TSP (TSP/Cit) and of Cit relative to Crn (Crn/Cit), respectively.

The first observation was that most of the data were consistent within some range and that there were also some occasional outliers, which we assigned as sample preparation mistakes occasionally induced by operators. 

Outlier spikes are present in all the graphs, with the highest deviations in the Crn/TSP graph and almost completely attenuated in the Crn/Cit graph, confirming the above-mentioned hypothesis on the various degrees of operator-induced variability for various types of integral referencing.

In terms of quantitative results, the overall variability Crn/ERETIC (Figure 12) considering all the operators and both instruments together was below 5% for 97.2% of all the data. When averaging the integrals for each operator separately, the overall variability was below 5% for 98.2% of all the data, and when averaging per operator and NMR instrument, the variability was below 5% for 97.9% of all the data and below 4% for 96.7% of all the data. When averaging per operator per NMR instrument and per recording day, the variability was below 5% for 98.2% of all the data, below 4% for 97.4% of all the data and below 3% for 94.3% of all the data.

The overall variability Cit/ERETIC (Figure 13) considering all the operators and both instruments together was below 6% for 96.9% of all the data and below 5% for 93.6% of all the data. When averaging the integrals for each operator separately, the overall variability was below 5% for 97.2% of all the data; when averaging per operator and NMR instrument, the variability was below 5% for 98.5% of all the data and when averaging per operator, NMR instrument and day, the variability was below 5% for 98.5% of all the data and below 4% for 97.4% of all the data. The results were in the same range of variability as Crn/ERETIC.

The overall variability TSP/ERETIC (Figure 14) considering all operators and both instruments together was below 10% for only 95.1% of all the data. When averaging the integrals for each operator separately, the overall variability was below 10% for 96.1% of all the data; when averaging per operator and NMR instrument, the variability was below 10% for the same 96.1% of all the data and when averaging per operator, NMR instrument and day, the variability below 10% was still unchanged at 96.1%. The results suggest that the operator-induced variability (at least for poorly trained operators) was higher than the overall instrumental-induced variability.

The overall variability Crn/TSP (Figure 15) considering all operators and both instruments together was below 10% for only 92.3% of all the data. When averaging the integrals for each operator separately, the overall variability was below 10% for 95.4% of all the data; when averaging per operator and NMR instrument, the variability was below 10% for the same 94.3% of all the data and when averaging per operator, NMR instrument and day, the variability below 10% was still unchanged at 94.3%. The results confirmed that the operator-induced variability was higher than the overall instrumental-induced variability.

The overall variability Cit/Crn (Figure 16) considering all operators and both instruments together was below 4% for all the data, below 3% for as much as 99.2% of all the data and below 2% for 95.1% of all the data. When averaging the integrals for each operator separately, the overall variability was below 2% for 97.7% of all the data, and when averaging per operator and NMR instrument, the variability was below 2% for 99.2% of all the data and below 1% for 97.7% of all the data. When averaging per operator per NMR instrument and per day, the variability was no longer improved, remaining below 2% for 97.7% of all the data and below 1% for 98.2% of all the data. The results confirmed the above assumption that reporting quantitative results relative to endogenous standards minimizes both the instrumental and operator-induced variability.

Thus, we may conclude that, when using this type of reporting, the metabolomics models may be over 96% accurate (below 4% variability) by employing any type of operator and can be easily improved to 98% (2% variability) by using reasonable trained operators without limiting the studies to specially trained operators. For most of the metabolomics studies, a 2% variation was not significant, as naturally occurring variability is much higher.

Coming back to Figure 12 and Figure 13, apart from the spikes induced by some sample preparation mistakes, a second observation was that some subtle patterns were visible within the main core of data.

When redisplaying the same data which is presented in Figure 12 for all Crn/ERETIC integrals, by only changing the order of the samples, then the main core patterns become more evident.

**Figure 17:**
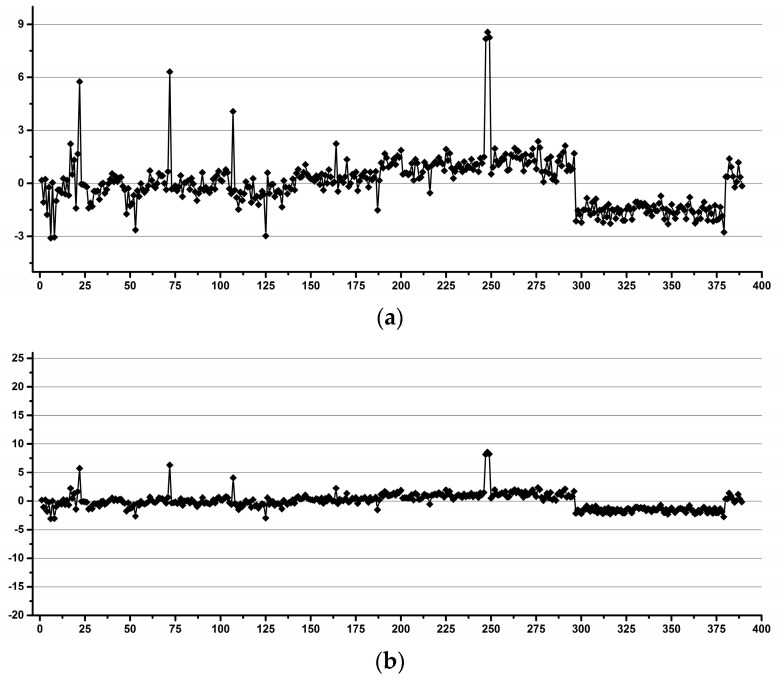
Crn/ERETIC integral variability relative to the averaged integrals for all samples. Samples are ordered by instrument used (I1 samples 1-188, I2 samples 189-389). Only 2.8% of all the data (i.e., 11 out of the total of 389 spectra) has an integral variability higher than 5% (±2.5% variability). Data scaled as previous figures for straightforward comparison (**b**) and data with higher amplification for better visualization (**a**).

Thus, in Figure 12, the data is ordered by operator for all instruments, and in Figure 17, the same data is reordered by instrument for all operators.

In order to assess if this pattern is a hardware-induced feature by one of the two instruments or by their environment disturbances, the variability was further calculated as the difference of the average for each instrument and not for all the data. 

**Figure 18:**
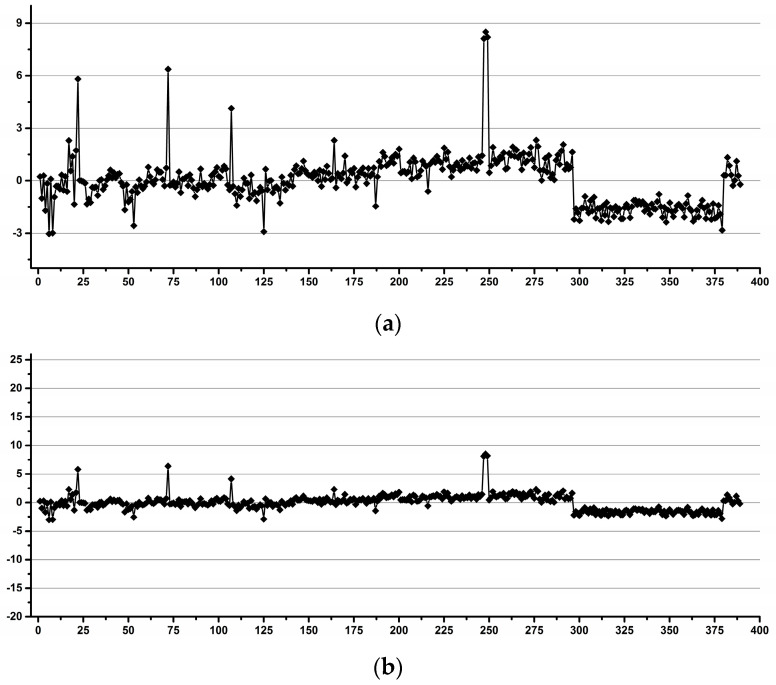
Crn/ERETIC integral variability relative to the averaged integrals for each instrument. Samples are ordered by instrument used (I1 samples 1-188, I2 samples 189–389). Only 2.8% of all the data (i.e., 11 out of the total of 389 spectra) has an integral variability higher than 5% (±2.5% variability). Data scaled as previous figures for straightforward comparison (**b**) and data with higher amplification for better visualization (**a**).

**Figure 19:**
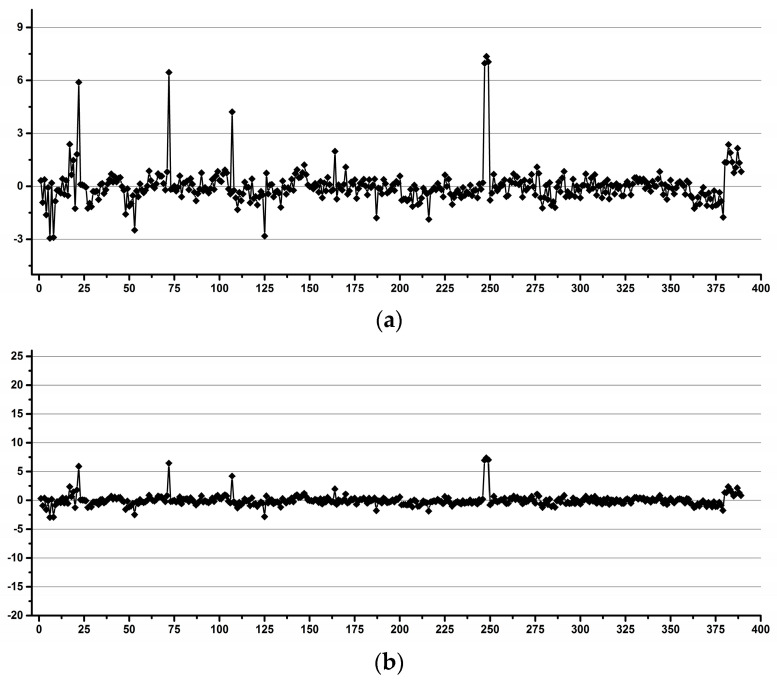
Crn/ERETIC integral variability relative to the averaged integrals for each instrument and recording day. Samples are ordered by instrument used (I1 samples 1-188, I2 samples 189-389). Only 2.8% of all the data (i.e.,11 out of the total of 389 spectra) has an integral variability higher than 5% (±2.5% variability). Data scaled as previous figures for straightforward comparison (**b**) and data with higher amplification for better visualization (**a**).

The results (Figure 18) indicate that the pattern is not instrument-dependent. Further reprocessing by day considering the differences in the average for each instrument and each day removed the pattern (Figure 19).

**Figure 20:**
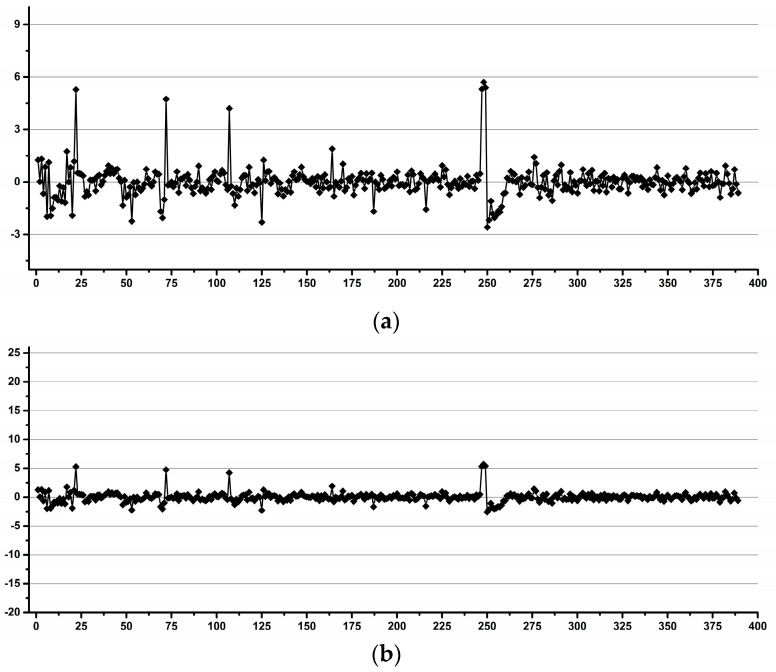
Crn/ERETIC integral variability relative to the averaged integrals for each instrument, recording day and each operator. Samples are ordered by instrument used (I1 samples 1-188, I2 samples 189-389). Only 2.8% of all the data (i.e., 11 out of the total of 389 spectra) has an integral variability higher than 5% (±2.5% variability). Data scaled as previous figures for straightforward comparison (**b**) and data with higher amplification for better visualization (**a**).

One persisting pattern in Figure 19 is associated with one of the operators, as further processing of the data as individual averages per operator removes all patterns (Figure 20).

Coming back to Figure 18, after a careful check of the recordings, the sudden change in the main core of data for the same instrument as the sample in position 189 was associated with a recalibration of the ERETIC signal based on the same QC sample that was used for all the data. 

This set of data clearly emphasized the variability induced by the ERETIC signal and separated this influence from the other instrumental and operator-induced factors. At this stage, it should be mentioned that the ERETIC-induced pattern had an overall variation below 4%, which was an accepted variation for the quantitation QC test specified by the NMR manufacturer for the IVDr methods. 

Thus, for variability below 4%, metabolomics data from different IVDr-ready instruments may be confidently used in the same statistical model, even if poorly trained operators are also employed.

The results were consistent within the same maximum 4% deviation for the NMR tube test experiment described above in Figure 8 and Figure 9 when the same solution of ethanol was run in the same tube several times with two different ERETIC calibrations. 

In order to push the variability of the data below 4%, internal referencing of the integrals (or concentrations) to an endogenous marker should be performed, as exemplified in Figure 16 when referencing the citrate relative to creatinine.

## 4. Conclusions

The results demonstrated that the current IVDr-ready NMR equipment allows entry-level operators and nonspecific NMR laboratories to obtain good-quality NMR metabolomics data with reasonable effort and based on an internal validation protocol, as we described here. The interlaboratory trial showed that, in a multipurpose NMR laboratory with NMR equipment often switched between multinuclear structure elucidation configuration and metabolomics-specific configurations and employing both poorly trained and metabolomics-specific trained operators, the resulting data may be confidently used in the same statistic model. Thus, the data may be exchanged between instruments (with similar hardware) from different laboratories and between operators with quite variable skills with a maximum of 4% variability. The limit may be further reduced below 2% by only employing internal sample referencing. These results were excellent for most of the metabolomics studies, as, usually, the nature-induced variability is well above 4%. Our study also demonstrated that, with the current state-of-the-art NMR equipment, reproducibility for metabolomics purposes may be further improved by using strictly selected NMR tubes, well-trained operators, fixed NMR configurations and strict SOP protocols. 

## Data Availability

All data were included in the Shewhart charts presented in the paper. All experimental details were given in the Section 3. Any further details may be obtained from one of the corresponding authors.
